# Intracranial pressure elevation post-stroke: Mechanisms and consequences

**DOI:** 10.3389/fstro.2023.1119120

**Published:** 2023-02-21

**Authors:** Rebecca J. Hood, Daniel J. Beard, Damian D. McLeod, Lucy A. Murtha, Neil J. Spratt

**Affiliations:** ^1^College of Health, Medicine and Wellbeing, University of Newcastle, Callaghan, NSW, Australia; ^2^Heart and Stroke Research Program, Hunter Medical Research Institute, New Lambton Heights, NSW, Australia; ^3^Acute Stroke Programme, Radcliffe Department of Medicine, University of Oxford, Oxford, United Kingdom; ^4^Department of Cardiology, School of Medicine and Health Sciences, Carl von Ossietzky University of Oldenburg, Oldenburg, Germany; ^5^Department of Neurology, John Hunter Hospital, New Lambton Heights, NSW, Australia

**Keywords:** intracranial pressure (ICP), cerebrospinal fluid (CSF), cerebral blood volume (CBV), cerebral edema, experimental stroke, collateral blood flow, early infarct expansion

## Abstract

Intracranial pressure (ICP) elevation post-stroke has long been thought of as a cause of secondary deterioration after large, malignant infarction, and dramatic ICP elevation is frequently a pre-terminal event. However, there is an increasing body of evidence to suggest that ICP also rises after small stroke, typically within 24 h of the infarct. The timing of this rise suggests that it may play an important role in the collateral failure associated with early infarct expansion. Despite its increasingly recognized importance to patient outcome, very little is currently known about the underlying mechanisms of ICP elevation post-stroke. The traditional understanding suggests ICP elevation occurs solely due to cerebral edema, however this does not seem to be the case in mild-moderate infarction. Instead, recent studies suggest a role for changes in cerebrospinal fluid (CSF) volume. In this article, we will discuss recent mechanistic observations, as well as the consequences of ICP elevation post-stroke.

## 1. Introduction

Acute ICP elevation (~24 h post-stroke) has been shown to occur in numerous animal models of stroke (Verlooy et al., 1990; Bell et al., 1991; Kotwica et al., 1991; Silasi et al., 2009; Wells et al., 2015; Sorby-Adams et al., 2019a,b; Thakkar et al., 2019). In 2012, our group recognized a link between minor stroke and dramatic, yet transient ICP elevation at 24 h post-stroke in rats (Murtha et al., 2014a). Previous studies by Kotwica et al. (1991), and Silasi et al. (2009) showed similar temporal profiles of ICP rise in rats, however the link between mild-moderate strokes and ICP was not a focus of their studies and was not specifically discussed (Kotwica et al., 1991; Silasi et al., 2009). Follow up studies from our group confirmed these findings in three different strains of rats (Murtha et al., 2014a, 2015, 2016; Beard et al., 2016a; Bothwell et al., 2021; Omileke et al., 2021a,b,c), using young and aged animals (Murtha et al., 2016) and, using transient (Murtha et al., 2014a, 2015, 2016; Omileke et al., 2021a,b,c) and permanent middle cerebral artery occlusion (MCAo) (Beard et al., 2016a) and, photothrombotic stroke (Beard et al., 2016a; Bothwell et al., 2021). This elevation was associated with larger infarct volumes and poorer functional outcomes. More recently, Alshuhri et al. (2020) showed similar findings in an additional strain of rats. The ICP elevation observed in the majority of these studies (20–30 mmHg above pre-stroke levels) is similar to that observed following large, hemispheric strokes in humans (Frank, 1995; Schwab et al., 1996).

Evidence of a similar ICP rise in higher order animals is scant and conflicting. Wells et al. (2015) showed that ICP was significantly higher than sham animals at 24 h after permanent and transient MCAo (2 h occlusion) in an ovine model of stroke. A recent study by Sorby-Adams et al. (2019b) showed a similarly timed significant ICP rise using a permanent MCAo model in sheep. However, when investigating the temporal profile of ICP after 2 h transient MCAo the same author found that ICP did not rise above sham levels until 5 days post-stroke (Sorby-Adams et al., 2019a). Evidence of early ICP elevation in other gyrencephalic non-human animals is limited by a lack of monitoring after 18 h post-stroke (Okada et al., 1983; Bell et al., 1991; D'Ambrosio et al., 2002; Toyota et al., 2002). It should be noted that, the stroke models used in the larger animal experiments necessitate opening the skull and dura, which may have the potential to modify an ICP response, particularly in the early post-operative period as is the case for any neurosurgical procedure requiring opening of the skull. These animal studies raise an important question—could a similar rise in ICP occur in patients with mild-moderate stroke?

In humans, ICP elevation is known to occur after large, malignant infarcts, typically between 2 and 5 days post-stroke, associated with edema and mass effect on imaging (Ropper and Shafran, 1984; Frank, 1995; Schwab et al., 1996). However, to our knowledge, there had been no clinical investigations of ICP in patients with mild-moderate stroke until recently. Such patients do not experience large volumes of edema, hence it had been assumed that ICP would not increase in these patients, and therefore highly invasive ICP monitoring was not justified. Many attempts have been made to develop accurate and reliable non-invasive methods for estimating ICP (for more information on invasive vs. non-invasive ICP methodologies please see review by Evensen and Eide, 2020). Measuring blood flow velocity with transcranial Doppler is currently the most extensively studied and the most widely available non-invasive method. Moreover, it is currently the only non-invasive method that can estimate ICP with a temporal resolution comparable to that of standard of care invasive methods. Using this technique, our group have recently assessed ICP in 10 patients with mild-moderate stroke (Kovacs et al., 2017). ICP increased significantly between 6 and 24 h among stroke patients (10.18 ± 4.25 mmHg 6 h, 13.31 ± 6.25 mmHg 24 h, *n* = 10*, p* = 0.02). There was no significant change in ICP among control patients (10.66 ± 3.24 mmHg baseline, 10.41 ± 2.81 mmHg 18 h later, *p* = 0.49, *n* = 75). In 7 of 10 stroke patients, ICP rose more than the 95th percentile among controls (1.2 mmHg), though their neurological deficits at the time of ICP measurement were mild (median NIHSS score of 3 at 24 h). Despite the modest rise in ICP compared with our animal studies, it must be noted that “baseline” ICP was measured at 6 h post-stroke. Bell et al. (1991) showed evidence that ICP may begin to rise as early as 4 h post-stroke in cats following experimental stroke. If the same is true for humans, the observed change in ICP from baseline values may be underestimated. Given the small number of patients assessed, more investigation is necessary to determine the range of ICP elevations after small strokes. However, even with an ICP rise of 5 mmHg, our animal data suggests that this may be enough to reduce flow through the penetrating arterial flow arising from collaterals and thus potentially lead to infarct expansion. Further, we only assessed ICP at the 24-h timepoint and so we do not yet know whether this rise is transient (as observed in our animal studies) or sustained. The importance of these findings is that they suggest that ICP elevation is not limited to large, malignant infarction. Furthermore, the timing of the ICP rise was before peak edema volume and at around the time of early infarct expansion. Importantly, this is the first evidence of ICP elevation in this patient population and challenges our current understanding of the prevalence and mechanisms governing ICP elevation post-stroke. Although extremely promising, more evidence is required to confirm the presence of an early ICP rise in mild-moderate stroke patients. If confirmed, our findings may justify ICP monitoring in this patient population.

## 2. Potential consequences of ICP elevation post-stroke

### 2.1. Collateral failure

There is now strong evidence from clinical studies that many patients with neurological deterioration in the first days after stroke suffer infarct expansion within the initially ischemic territory, rather than recurrent thrombosis. The infarct expansion is associated with failure of collateral blood flow (Coutts et al., 2012; Campbell et al., 2013). Leptomeningeal collateral vessels are anastomotic connections between the distal ends of adjacent arterial territories and supply residual retrograde perfusion to the ischemic territory in the event of arterial occlusion during ischemic stroke. This is the mechanism for residual perfusion, which may maintain some of the ischemic tissue for some time after vessel occlusion. This threatened, but potentially salvageable tissue is known as the ischemic penumbra (Astrup et al., 1981). It surrounds the already irreversibly damaged infarct core, and its survival is a product of residual perfusion, and time. The recent positive clinical trials of endovascular therapy in the late time-window have now shown that in those with excellent collaterals and small initial infarct core, penumbra may survive for at least 24 h (Albers et al., 2018; Nogueira et al., 2018). With the more widespread use of advanced brain imaging, it has been recognized that patients with rapidly improving neurological deficits may have persistent large vessel occlusion, with excellent collateral supply (Coutts et al., 2012). However, it is also clear that such patients may be at significant risk of in-hospital deterioration. Initially excellent collateral vessel flow can subsequently fail, leading to expansion of the infarct into penumbra and early neurological deterioration (Dávalos and Castillo, 2001; Asdaghi et al., 2011; Coutts et al., 2012; Campbell et al., 2013). There is not yet any definitive evidence for the mechanism of such collateral failure. Some hypotheses have been advanced, such as collateral vessel thrombosis (Liebeskind, 2003), reverse Robin Hood Syndrome (Alexandrov et al., 2007), and venous steal (Pranevicius et al., 2012), without much direct evidence for any, to date. We believe that reduction of cerebral perfusion pressure (CPP) due to ICP elevation, is a more plausible explanation, and for which there is now an increasing body of experimental, and preliminary clinical evidence.

CPP is the driving force for cerebral blood flow (CBF). Because of the “closed” skull, and unlike in all other vascular beds, there is a significant “downstream pressure,” the ICP, which influences cerebral perfusion in addition to blood pressure (expressed as mean arterial pressure; MAP). This is expressed in the equation (Urrutia and Wityk, 2008):


$$\begin{array}{l} CPP=MAP-ICP \end{array}$$

Under normal circumstances in healthy brain, cerebral autoregulation preserves tissue perfusion despite fluctuations in CPP. The cerebral vessels are in a partial state of constriction, from which they can increase or decrease diameter and hence regulate CBF in response to metabolic demand, to ensure constant supply of blood flow to the brain over a wide range of CPP. The primary determinant of this basal vessel tone is myogenic behavior of the vascular smooth muscle cells that constrict in response to increased CPP or dilate in response to decreased CPP to maintain a constant CBF (Johansson, 1989). In the normal non-ischemic brain, where autoregulation is intact, an alteration in MAP that changes CPP values within the range of 50–150 mmHg will have little to no effect on CBF (Novak et al., 2004). However, in the ischemic cortex during stroke, cerebral autoregulation is severely impaired since vessels are already maximally vasodilated in response to ischemia. Hence, CBF becomes CPP dependent, and any reduction in CPP will preferentially reduce flow to the penumbra, while autoregulation preserves flow to the non-ischemic brain. This may give rise to what has been termed the “reverse Robin Hood” phenomenon, whereby vasodilators, given in an attempt to improve collateral flow, may lower blood pressure, and hence CPP, thus “stealing” blood flow from the penumbra, where the need is greatest (the poor), and increasing flow to other non-ischemic brain regions (the rich), which already have plenty (Alexandrov et al., 2007). Note that mechanisms such as inflammation (Eldahshan et al., 2019), alterations in energy metabolism (Ferrari and Villa, 2022) may also impair autoregulation. Readers are directed to Claassen et al. (2021) for a compressive review of autoregulation physiology and pathophysiology.

It has previously been hypothesized that patients with early infarct expansion may have fluctuations in blood pressure due to autonomic dysfunction (Palamarchuk et al., 2013). However, despite blood pressure being measured regularly in almost all hospitalized stroke patients, evidence for the role of drops in blood pressure preceding deterioration is lacking. The role of the other side of the CPP equation, ICP, is less often discussed. We have shown that transient ICP elevation over some hours, around 24 h after seven minor stroke, is a near universal phenomenon in rodent stroke models (Murtha et al., 2014a, 2015, 2016; Beard et al., 2016a; Alshuhri et al., 2020; Bothwell et al., 2021; Omileke et al., 2021a,b,c). Additionally, we have shown that artificially elevating ICP to similar levels that occur after minor experimental stroke immediately decreases leptomeningeal collateral blood flow and blood flow to the penetrating arterioles they supply (Beard et al., 2015). This provides evidence, first, that ICP elevation may be a feature of minor stroke, and second, it establishes the biological plausibility of such an elevation causing reduction (“failure”) of collateral flow of sufficient magnitude to cause infarct expansion and early neurological deterioration in some stroke patients (previously reviewed in Beard et al., 2016b).

### 2.2. Reperfusion CBF deficits

**Figure 1:**
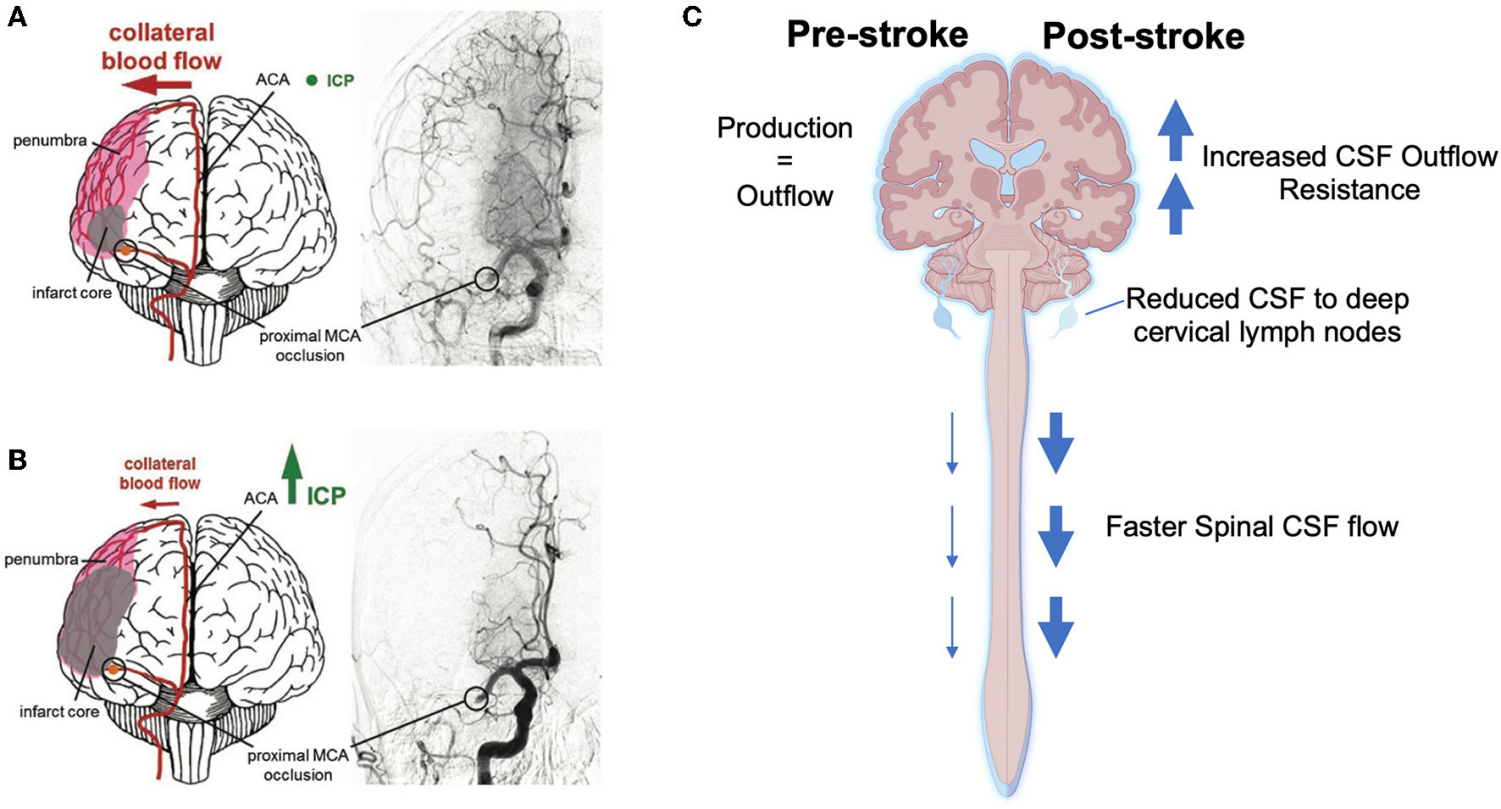
Current hypotheses of the cause and consequence of intracranial pressure (ICP) elevation after mild-moderate stroke. **(A)** Collateral perfusion helps to limit the extent of the ischemic core after stroke if ICP remains within the physiological range. **(B)** Increasing ICP can dramatically reduce collateral blood flow after ischemic stroke. **(C)** Schematic showing changes to the cerebrospinal fluid (CSF) system post-stroke that may result in increased CSF volume and thus increased ICP. ACA, anterior cerebral artery; MCA, middle cerebral artery. Part **(A, B)** were reprinted from Demuth et al. (2017). Copyright (2017), with permission from IOS Press (96). The publication is available at IOS Press through http://dx.doi.org/10.3233/RNN-160690. Part **(C)** was created with BioRender.com.

Autoregulatory failure begins due to a drop in intravascular pressure and maximal vascular dilation, however there may be other factors that become important over time that may result in autoregulatory deficits even after reperfusion, and may last for up to 2–3 weeks after stroke (Olsen, 1986; Macfarlane et al., 1991). For example, it has been shown that the loss of myogenic tone in the middle cerebral artery extracted from rats undergoing MCAo (Cipolla et al., 2001; Cipolla and Curry, 2002) is positively associated with the degree of loss of the actin cytoskeleton in smooth muscle cells surrounding the isolated arteries, essentially paralyzing the smooth muscle until new actin can be formed (Cipolla et al., 2001). Therefore, ICP elevation in combination with persistent autoregulatory failure may lead to perfusion deficits in the ischemic hemisphere following reperfusion. We have shown in two separate studies that temporary MCAo results in higher ICP elevation at 24 h (42 ± 7 mmHg) (Murtha et al., 2014a, 2015), compared to permanent stroke (17 ± 7 mmHg) (Beard et al., 2016a). Such a dramatic ICP rise, if not accompanied by a rise in blood pressure, might have quite consequential effects on perfusion, particularly in the most vulnerable regions of the reperfused penumbra, in which autoregulation would also be expected to be most impaired. This may be further exacerbated by the non-linear effect of ICP elevation on perfusion to penetrating arterioles that branch off from the pial branches of the middle cerebral artery. We have shown that ICP elevation to as little as 5 mmHg above baseline, almost completely abolished penetrating arteriole flow in the watershed territory (Beard et al., 2015). This is not surprising as penetrating arterioles are known as the “bottleneck” of perfusion to the cortex and have been shown to maintain basal tone even after 2 h of temporary MCAo and 30 min of reperfusion (Cipolla et al., 2013). This raises the possibility that some of the injury that has in the past been attributed to “reperfusion injury” may have been caused by ICP elevation-induced reduction in CBF, within reperfused penumbra (Aronowski et al., 1997) (Figures 1A, B).

### 2.3. ICP rise is a potential physiological stimulus for infarct expansion after reperfusion

The diffusion-weighted (DWI) lesion on magnetic resonance imaging (MRI) is the earliest marker of infarction after stroke but is known to “reverse” to greater or lesser extent with early reperfusion (van Lookeren Campagne et al., 1999; Labeyrie et al., 2012; Asdaghi et al., 2014; Soize et al., 2015). However, initial excitement about this possibility abated significantly when it was recognized that DWI lesions tended to re-expand at imaging time points beyond 24 h. This was interpreted as indicating that the initial reversal represented imaging artifact (van Lookeren Campagne et al., 1999; Campbell et al., 2012). This conclusion was reinforced by the lack of clinical deterioration associated with DWI lesion recurrence. However, we now know that at the time DWI lesion recurrence occurs (in rats and humans), there is a newly identified physiological stimulus (ICP rise) that causes blood flow reduction in the relevant tissues, in experimental models (Beard et al., 2015). The timing also fits with that of neurological deterioration in patients with early infarct expansion. We propose that the severity of insult sufficient to cause initial (reversible) DWI change is similar to that required for both neurological functional and blood vessel autoregulatory impairment. Hence DWI lesion recurrence may be due to infarction in vulnerable tissue experiencing secondary ischemia. Such regions would be expected to remain functionally silent at such an early time point, since recovery of neurological function is known to occur over at least days, after significant ischemia, which explains why there is no apparent neurological deterioration. However, this would still be clinically important, if shown to be true, since such patients will not recover to the full extent that would have been possible, had they not experienced a secondary ischemic insult due to ICP elevation.

### 2.4. Drops in CPP may provide a unifying theory of selective watershed infarction during carotid artery occlusion

**Figure 2:**
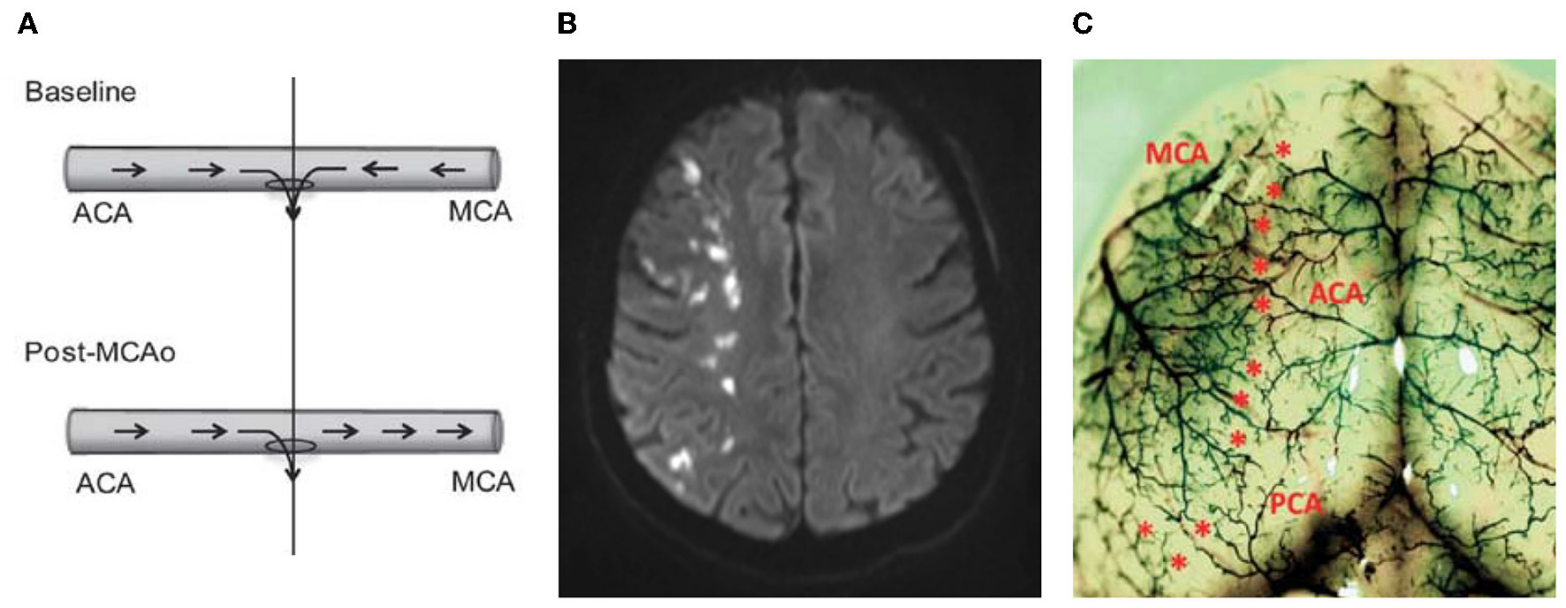
Hypothetical role of leptomeningeal collaterals in watershed infarction. **(A)** Schematic of collateral blood flow direction before and after middle cerebral artery occlusion (MCAo). The “watershed” penetrating arteriole at the confluence of the bidirectional collateral vessel blood flow (top panel) was used to demarcate the anterior (ACA) and middle (MCA) cerebral artery portions of the vessel (vertical line). At baseline blood flows in two directions (from the ACA and the MCA) and both flowing into the midpoint penetrating arteriole to supply blood to the watershed territory. **(B)** Human DWI-MRI showing so called “string of pearls” watershed infarction—each “pearl” represents the territory of a single watershed penetrating arteriole, at the junction of 2 major arterial territories (e.g., anterior-middle cerebral arteries watershed). **(C)** Rat cortical vasculature showing the location of pial collateral vessels (colored latex perfusion). Collaterals between the ACA and the MCA, and posterior cerebral artery and MCA are marked with asterisks. Note the striking similarities between the distribution of cortical collaterals seen in the rat and the distribution of the string of pearls sign in the watershed region shown in **(B)**. Part **(A, C)** reprinted by Permission of SAGE Publications. Beard et al. (2015), copyright © 2015 by SAGE Publications.

Work by us and others has demonstrated that blood flow is present in collaterals under basal conditions and after reperfusion in rats (Toriumi et al., 2009; Beard et al., 2015). Leptomeningeal collaterals receive a dual supply of blood from the two territories they connect. Under normal conditions, the slow turbulent flow from each territory meets and enters the watershed penetrating arteriole at the midpoint of the collateral vessel, to perfuse the underlying watershed territory (Figure 2A). The small diameter, high resistance penetrating arteriole arises off a much larger vessel. It is thus exposed to greater pressure than would normally be the case for a typical vessel of this size, that arises only after multiple progressively smaller branching vessels, with progressively greater resistance and thus pressure drop. In this regard, these vessels are perhaps most analogous to the deep perforating vessels arising from the circle of Willis, although the latter are exposed to the even higher pressures of the proximal intracranial arteries. Also of note, the midpoint, or watershed, is defined by the point at which pressures from each side equalize. Thus, in the presence of intracranial stenosis affecting one territory, the midpoint/watershed will move toward the affected territory, as pressure within that territory drops, due to the increased resistance created by the stenosis. This unique baseline flow arrangement may mean that small reductions in CPP will have a much greater effect on “watershed” penetrating arterioles, since a greater driving pressure is required to maintain flow across high resistance vessels. This may be a potential explanation for why the external or cortical watershed territory is prone to infarction (“string of pearls” sign on MRI) following occlusion of the internal carotid artery (ICAo) in the setting of poor Willisian collateral flow (Hendrikse et al., 2001; Momjian-Mayor and Baron, 2005) (Figure 2B). This “string of pearls” pattern of infarction shows a strikingly similar distribution to the location the leptomeningeal collaterals and associated penetrating arterioles (Figure 2C). If perfusion to the watershed is “teetering on the edge,” then even the slightest reduction in perfusion pressure (e.g., following ICAo, lowering local arterial pressure, or from ICP elevation) may reduce watershed perfusion below the threshold for infarction. This may explain why human imaging studies have shown reduced perfusion in the cortical watershed territory corresponding to the area of infarction following ICAo (Leblanc et al., 1987, 1989). These findings have formed the basis for the “hemodynamic failure” hypothesis for cortical watershed infarction.

Alternatively, other studies have reported the presence of microembolic signals using transcranial Doppler (Siebler et al., 1992) and the presence of microemboli in the distal arterial tree (Torvik and Skullerud, 1982) which are associated with cortical watershed infarction following ICAo. It is presumed that the microembolic signals on transcranial Doppler indicate embolic materials within the insonated artery, originating from the ICA thrombosis (Sitzer et al., 1995). Embolic material of specific size (150–210 μm) are thought to be preferentially distributed in the distal arterial tree (cortical watershed) as a result of the Fahraeus-Lindqvist (plasma skimming) effect; which states that “the viscosity of a suspension of particles flowing through bifurcations will change, such that one of the branches has a suspension of particles that is less viscous than the parent trunk” (Pollanen and Deck, 1990). This means that large embolic material, which travel in the axial stream of flowing blood, will have little deviation from the parent vessel at asymmetric branch points, while the marginal plasma flow with fewer emboli gets skimmed off at each branch point. This ultimately funnels the embolic material to the distal arterial tree (Pollanen and Deck, 1990). These findings have formed the basis for the “embolic” hypothesis for cortical watershed infarction. The watershed arterioles are particularly susceptible, since it is at this point that the flow from the 2 arterial territories meet, and the embolus may act like a “plug” at the origin of the small diameter draining penetrating arteriole, through which flow from both territories drains (Figure 2A).

Our previous work showing the extreme susceptibility of flow at the watershed penetrating arterioles to even minor change in ICP, and hence CPP, may provide a unifying theory for both the hemodynamic failure and embolic theories of watershed infarction. The already slow blood flow velocity in the collaterals combined with ICAo may further reduce blood flow velocity (hemodynamic failure theory) to levels conducive to *de novo* thrombosis and embolization (embolic theory) within the leptomeningeal collateral/cortical watershed region (Powers, 1991). The bidirectional blood flow may then wash the emboli into the central watershed penetrating arteriole (like a sink) leading to penetrating arteriole occlusion and subsequent watershed infarction. We believe this theory warrants further investigation.

## 3. Mechanisms of post-stroke ICP elevation

ICP elevation can be attributed to an uncompensated increase in the volume of one or more of the intracranial contents as outlined by the Monro-Kellie doctrine. In short, this doctrine states that the intracranial compartment is a closed system within the non-expandable, rigid skull made up of three components: tissue (including edema), blood and CSF (Monro, 1783; Kellie, 1824; Burrows, 1846). An increase in any one of the components leading to an increase in volume, will result in a decrease in one or both other components, to maintain constant ICP. However, the intracranial cavity only has a small reserve for accommodating additional volume, which, once exhausted, will result in a dramatic rise in ICP.

### 3.1. Tissue volume

Up until recently, post-stroke ICP elevation was generally considered to result from cerebral edema (Ropper and Shafran, 1984; Schwab et al., 1998). There are 3 categories of edema: cytotoxic, ionic and vasogenic. Cytotoxic edema refers to the excess accumulation of fluid within the intracellular space. During a stroke, cerebral cytotoxic edema occurs as a result of the failure of ion pumps during the ischemic cascade. The cellular influx of calcium, sodium and chloride exceeds the potassium efflux and the subsequent water accumulation results in swelling of the affected cells (Simard et al., 2007). Cytotoxic edema occurs within minutes to hours after stroke and is worsened by spreading depolarizations, developing locally within a few seconds after the onset of the ischemia (for more information see review Dreier et al., 2018). Importantly, cytotoxic edema is potentially reversible if energy metabolism is restored. However, excessive water accumulation has the potential to cause cellular rupture. Cytotoxic edema alone does not influence ICP, as it is merely a redistribution of water (and ions) from the extracellular to the intracellular space. However, this redistribution creates gradients promoting influx of ions and water from capillaries into the interstitial space, termed ionic edema. This does increase the net intracranial volume and thus results in ICP elevation. As the extracellular sodium ions are drawn from the extracellular space to the intracellular space an osmotic gradient is formed which favors the influx of intravascular sodium and water into the extracellular space causing tissue swelling (Mori et al., 2002). A recent study by Mestre et al. (2020) has shown evidence of CSF redistribution from glymphatic circulation within minutes of stroke suggesting that CSF may also contribute to the influx of water into the extracellular space. Importantly, for ionic edema to occur there needs to be a source of fluid, i.e., blood/ glymphatic flow (Simard et al., 2007). Therefore, ionic edema is more likely to occur in the penumbra than the infarct core. Vasogenic edema occurs in the days following stroke and involves the breakdown of tight junctions between endothelial cells that make up the blood-brain-barrier (for more information on the underlying mechanisms see review by Stokum et al., 2016). This allows the passage of plasma proteins and fluid into cerebral tissue leading to increased extracellular fluid volume. The resulting swelling has the potential to displace brain structures and even hemispheres, leading to the compression of neurons and cerebral blood vessels.

Much of our understanding of ICP elevation in patients post-stroke comes from patients with malignant infarction. In this patient population, ICP elevation is associated with large infarction, significant cerebral edema and mass effect, peaking 3–5 days post-stroke (Ropper and Shafran, 1984; Frank, 1995; Hacke et al., 1996; Schwab et al., 1996). Extremes of ICP are often a pre-terminal event in such patients, however notably, clinical signs indicating herniation from mass effect are normally present before the pre-terminal dramatic ICP rise (Schwab et al., 1996; Poca et al., 2010), suggesting the latter is an effect of herniation (likely mediated by blockage of venous outflow) rather than the cause thereof. Similarly, while edema is present in all such patients (and the reason ICP is being monitored), only a minority have significant ICP rise prior to signs of herniation (Frank, 1995).

There is an increasing body of preclinical evidence suggesting that stroke induced ICP elevation can be caused by other mechanisms, in addition to edema. When investigating potential mechanisms of the dramatic ICP elevation observed at 24 h post-stroke in rats with mild-moderate infarcts, we observed that significant ICP elevation occurred in the presence of small infarcts with minimal to no cerebral edema (Murtha et al., 2014a, 2015, 2016). This finding was met with considerable skepticism since it ran counter to the general understanding of post-stroke ICP elevation. To ensure the veracity of the findings, they were confirmed using histology, wet weight-dry weight and *in vivo* magnetic resonance imaging to measure edema (Murtha et al., 2015). A recent study from Alshuhri et al. (2020) supports and extends these findings, showing no correlation between edema and ICP elevation 24 h after permanent MCAo in rats. Furthermore, in Sprague-Dawley rats, our group demonstrated that treatment with short duration hypothermia soon after stroke completely prevented ICP elevation at 24 h despite the presence of large edema volumes (Murtha et al., 2015).

There are many similarities in the biological processes recruited in response to many acute neurological diseases. It is enticing to speculate that this may also apply to other acute neurological injuries associated with ICP elevation.

### 3.2. Cerebral blood volume

Changes in cerebral blood volume (CBV) may contribute to ICP elevation post-stroke, however, no studies have simultaneously monitored ICP with a continuous measurement of CBV following stroke. There are data, however, in two patients with intracranial hypertension, where acute transient rises in ICP (plateau waves) were simultaneously recorded with continuous regional CBV (rCBV) measurements from multiple brain locations (Risberg et al., 1969). Here, it was shown that every plateau wave was accompanied by an increase in rCBV, after the initial spontaneous ICP elevation. Interestingly, the largest changes in rCBV were measured from parts of the brain that included the ventricles and the choroid plexuses. These data suggest that there may be an important role for CBV in some forms of ICP elevation, but its investigation has been limited by the lack of well-validated tools to measure it. Therefore, the specific role of CBV during ICP elevation in stroke and other neurological disorders awaits further investigation.

### 3.3. Cerebrospinal fluid volume

Accurately quantifying CSF volume has proven challenging, both in humans and in experimental animals. Measurement is complicated due to the space being surrounded by bone, the constant yet uneven circulation of CSF, and the convoluted pathways CSF travels around the brain and spinal cord. Some have sought to measure volumes post stroke, e.g., using ventricular volume as a surrogate measure (Dhar et al., 2016, 2018, 2020, 2021; Kauw et al., 2019, 2022; Monch et al., 2020; Jiang et al., 2022), but given the ventricular compression known to occur from hemispheric swelling, this is not an entirely satisfactory surrogate for total cranio-spinal CSF volume. Similarly, CSF tracer dilution studies have been used to try to estimate volume (Oshio et al., 2005). Such studies rely on achieving steady-state tracer concentration. However, the convoluted CSF flow pathways and, speed of turnover likely prevent this ever occurring within the entire CSF space, making such methods unreliable (Oreskovic and Klarica, 2014). Another important consideration for CSF volumetric analyses is that CSF volume is not confined within the cranial cavity. Investigations into CSF volume often focus on cranial CSF (Chiu et al., 2012; Murtha et al., 2014b) due to the technical challenges of spinal CSF imaging, and thus likely underestimate total CSF volume.

One way to circumvent these issues is to use surrogate measurements of CSF volume, e.g., *via* changes in CSF dynamics (production, flow, and outflow) (Heisey et al., 1962; Pappenheimer et al., 1962; Yasuda et al., 2002; Karimy et al., 2015; Bothwell et al., 2021). CSF volume is a product of the balance between the production and outflow of CSF. Therefore, increased CSF production and/ or decreased outflow that results in a change in CSF volume could cause ICP to rise. Using this understanding, there is an increasing body of pre-clinical evidence implicating the CSF system in the early ICP rise post-stroke (Figure 1C). Alshuhri et al. (2020) investigated CSF production and outflow in rats post-stroke. Whilst they did not detect an increase in CSF production, the authors found evidence of a direct link between CSF outflow resistance and ICP post-stroke. The authors showed a 2-fold increase in CSF outflow resistance at 24 h post-permanent MCAo in rats which significantly correlated with ICP. These findings suggest that CSF volume may be contributing to post-stroke ICP rise, and that reduced or impaired clearance of CSF could be an underlying mechanism. The specific cause of the increased outflow resistance was not investigated in those studies, however Bothwell et al. (2021) found evidence that stroke reduces transit of CSF tracers to the deep cervical lymphatics (one of the CSF outflow pathways) 18 h after photothrombotic stroke in rats with ICP rise. Interestingly, an increase in spinal CSF transit was observed by the authors, suggesting the possibility that this outflow pathway may be a compensatory mechanism in response to elevated ICP when direct cranial clearance pathways are absent. Interestingly, similar reductions in transit of CSF tracers to cervical lymphatics have been observed in rats (Bolte et al., 2020) and rabbits (Griebel et al., 1989), after experimental traumatic brain injury and subarachnoid hemorrhage, respectively. This may indicate that there are similar mechanisms present in other acute neurological disorders that involve raised ICP.

## 4. Conclusions and future directions

This review highlights compelling evidence for a previously unsuspected early, transient elevation of ICP occurring ~24 h after the onset of minor/moderate stroke. In experimental studies, greater ICP elevation was seen after temporary than permanent vessel occlusion. Increasing experimental evidence suggests a role for reduction of CSF outflow, thereby increasing CSF volume, as a contributing factor to stroke-induced ICP rise, however the exact mechanism is yet to be elucidated. Regardless of the cause, it has become clear that ICP changes may be frequent and dynamic and ICP elevation may be an important, previously unrecognized, pathophysiological mechanism of infarct expansion and neurological deterioration in stroke patients. Taken as a whole, the findings highlight how little is known about the fundamental mechanisms of ICP regulation in health and disease. Future studies should be undertaken to confirm the clinical relevance of ICP elevation in patients with mild-moderate strokes. If confirmed this may have major implications for patient management, including the justification for ICP measurement in this population.

## Author contributions

The manuscript was written by RH, DB, DM, and NS. Figures were constructed by RH and DB. All authors contributed to the conception of this review. All authors edited and approved the final manuscript.
